# Misdiagnosis of peripheral abscess caused by duodenal foreign body: a case report and literature review

**DOI:** 10.1186/s12876-020-01335-7

**Published:** 2020-07-23

**Authors:** Zhihui Wang, Zhiqiang Du, Xiangrong Zhou, Tianming Chen, Chunyan Li

**Affiliations:** Department of Gastroenterology, Jianyang People’s Hospital, No. 180 Yiyuan Road, Jianyang City, 641400 China

**Keywords:** Duodenal, Foreign body, Abscessus, Fishbone, Case report

## Abstract

**Background:**

The induction of chronic inflammation, perforation, and abscess by foreign bodies (FBs) in adults is uncommon. We present a delayed diagnosis case for a patient who had a fishbone stuck in the duodenal bulb, resulting in chronic abdominal pain for nearly 3 months. We present the diagnosis and treatment procedures for chronic patients, which differ from those for acute and emergency FB ingestion, and also summarize the characteristics of such patients through a systematic literature review.

**Case presentation:**

A 68-year-old woman was brought to our hospital with repeated right upper abdominal pain lasting for 3 months and aggravation for 9 h. Computed tomography (CT) showed a streaky high-density shadow (approximately 3 cm in length) on the posterior wall of the gastric antrum extending outside the wall. Endoscopic ultrasonography showed hyperechoic space with a cross-section of approximately 0.1 × 0.1 cm in the deep submucosal layer of the local stomach, accompanied by an acoustic shadow in the rear. The possibility of a fishbone as well as perforation was considered and the object was removed using FB forceps. Fasting as well as acid inhibition and anti-infection medication were prescribed for the patient. She eventually recovered and was discharged from the hospital.

**Conclusion:**

Endoscopic intervention can be recommended as the first option for patients with gastrointestinal FBs.

## Background

The ingestion of foreign bodies (FBs), including food, is a common incidence in clinics. Although most cases of FB ingestion occur in children, a moderate proportion occurs in adults during dining. Patients with psychiatric disorders, mental retardation, alcohol intoxication, and other impairments have a higher risk of ingesting FBs [1]. According to the reported statistics, FB ingestion-related deaths are as high as 1500 cases each year in the US [2], and retrospective analyses in Asian populations have increasingly reported cases of FB ingestion [3–5]. Thus, FB ingestion in both children and adults has emerged as a growing healthcare challenge as well as a medical burden both worldwide and in Asia.

Nearly 80–90% of FBs naturally pass through the gastrointestinal tract spontaneously without discomfort but those that cause significant clinical symptoms often require medical intervention [6]. For most emergency patients that visit the hospital, FBs in the upper gastrointestinal tract, especially the upper one-third of the esophagus, may lead to serious complications, and even cause death in high risk patients with a history of gastrointestinal tract surgery or gut malformations [7]. Without appropriate treatment, the prognosis of FB ingestion is poor because of the risk of various complications such as cholangitis, liver abscess, peritonitis, pancreatitis, and cholecystitis, with a small proportion (< 1%) leading to perforations and obstruction [8].

Endoscopy and surgery are the major choices of treatment for FB ingestion, and can be successfully performed for nearly all patients [9, 10]. However, accurate diagnosis of FB ingestion is imperative. Unlike with acute patients, the diagnosis of FB ingestion is often challenging because patients are frequently unaware of ingesting FBs, and the clinical manifestations range from no symptoms to nonspecific abdominal pain, nausea, vomiting, or fever. In this paper, we present such a case and also summarize the characteristics of such patients through systematically reviewing related literature.

## Case presentation

### Basic information

A 68-year-old woman was brought to our hospital with repeated right upper abdominal pain lasting for 3 month and aggravation for 9 h. From February 2019 to May 2019, the patient experienced multiple episodes of dull epigastric pain and discomfort, which was often aggravated in the morning with paroxysmal colic. The patient had visited many hospitals without symptom control, but had improved after orally taking omeprazole and anti-inflammatory agents. She did not have black stools or similar symptoms before the symptoms appeared 3 months earlier.

### Routine examination and treatment

**Fig. 1:**
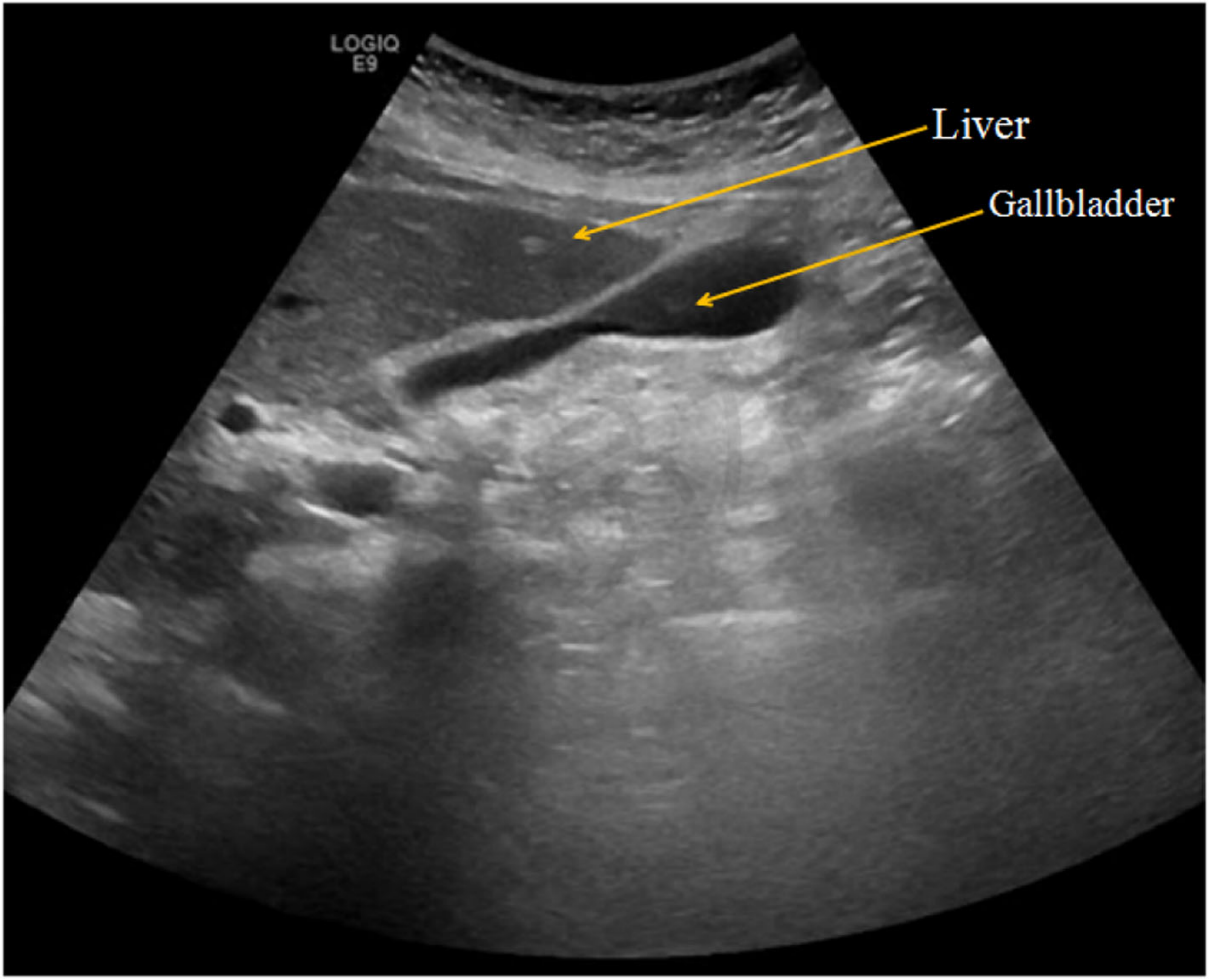
B-ultrasonography examination indicated no obvious abnormalities in the liver, gallbladder, pancreas, spleen, and kidney

**Fig. 2:**
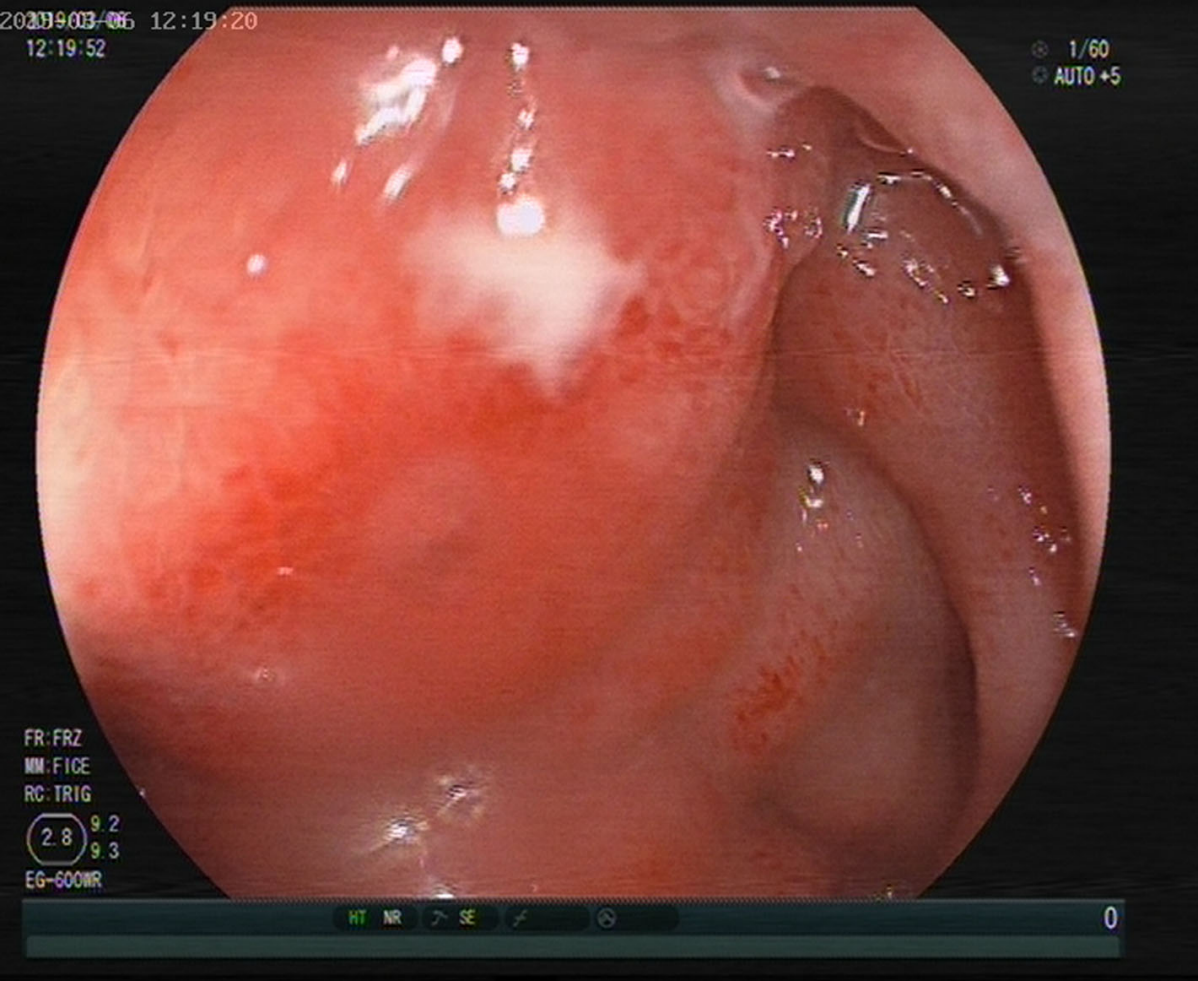
Gastroscopy examination showed obvious hyperemia and edema in the anterior wall of the duodenal bulb, superficial white pus coating on the surface, and semicircular swelling of the mucous membrane in the cavity

Physical examination showed obvious tenderness in the lower right epigastric region of the xiphoid process, and there was no rebound pain or muscle tension. Blood examination only indicated slightly elevated levels of C-reactive protein, while other tests including routine blood tests were normal. Chest X-ray and B-ultrasound indicated no obvious abnormalities, as shown in Fig. 1. Gastroscopy revealed obvious hyperemia and edema in the anterior wall of the duodenal bulb, with superficial white pus coating on the surface, and semicircular swelling of the mucous membrane into the cavity, as shown in Fig. 2.

A duodenal bulbous bulge with bulbous inflammation (possibly due to external pressure on the gall bladder abscess) and/or duodenal bulb ulcer were first considered to be responsible for the discomfort. Cefazoxime sodium was intravenously administered at 2 g and Q12h. Omeprazole was orally administered at 40 mg and Q12h. However, the effect of the anti-ulcer therapy was unremarkable as the symptoms were not significantly relieved and epigastric pain was still present. Additionally, the paroxysmal spasmolysis could not be controlled by intramuscular injection of anisodamine.

### Further examination and confirmation

**Fig. 3:**
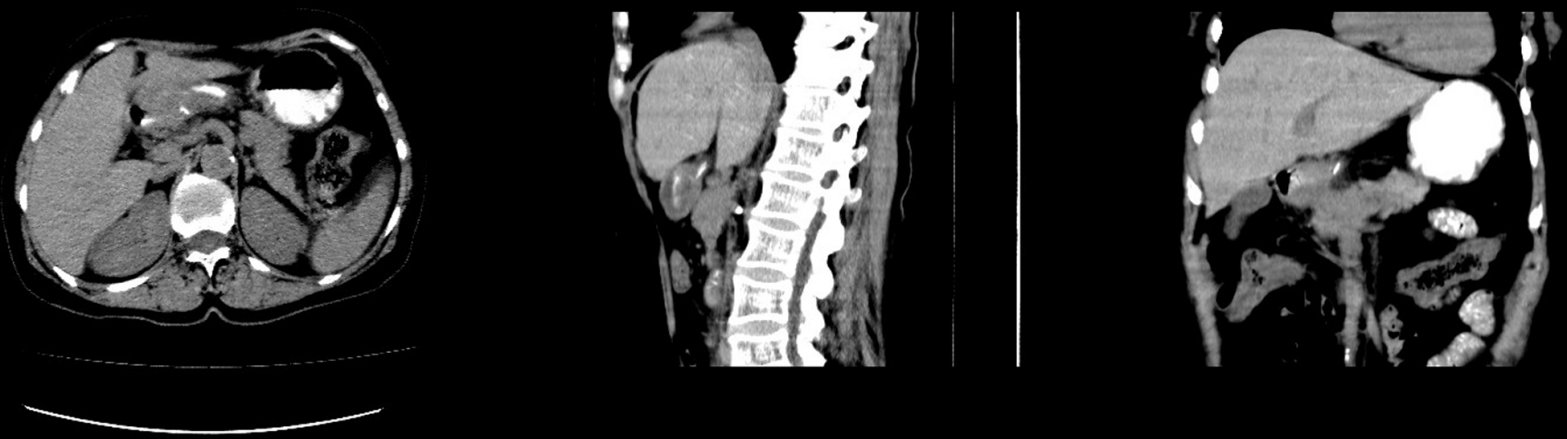
CT scan showed a streaky high-density shadow on the posterior wall of the gastric antrum extending outside the wall, with a length of about 3 cm

**Fig. 4:**
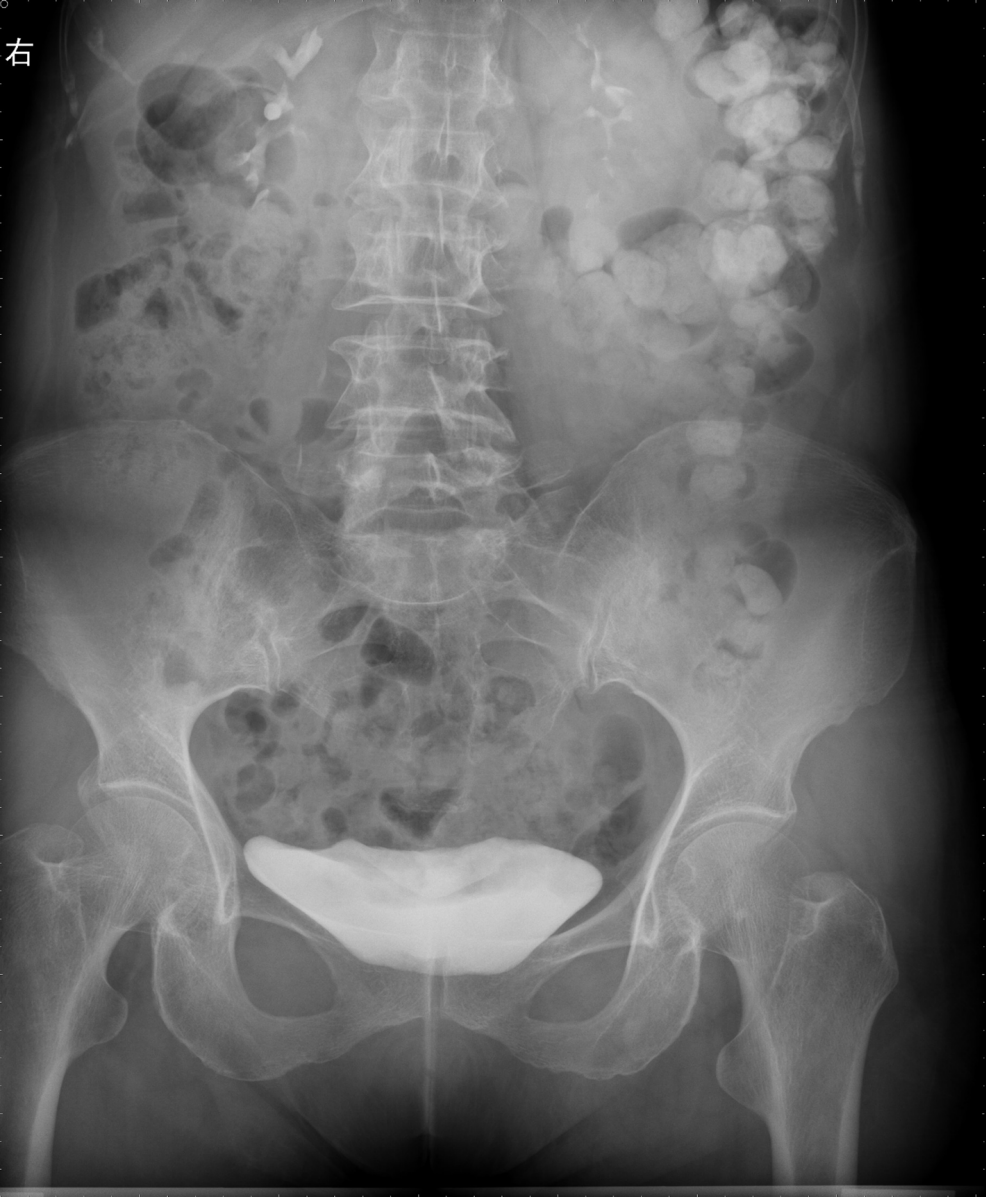
Abdominal X-ray imaging suggested that there was a dense shadow in the duodenal bulb with a length of about 2.7 cm, which might be considered a foreign body

**Fig. 5:**
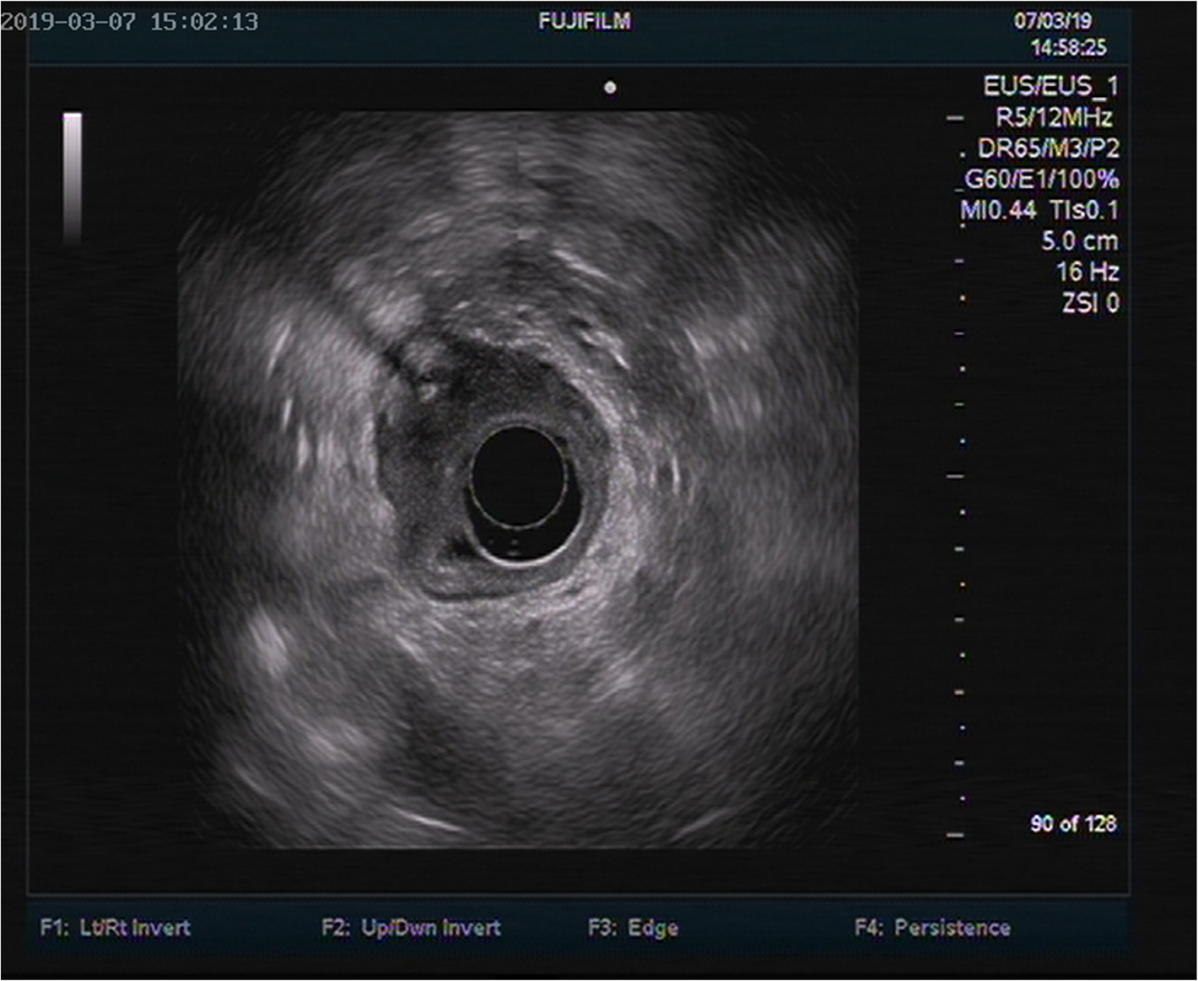
Ultrasound gastroscopy showed that the anterior wall of the duodenal bulb was obviously protruding into the cavity, a superficial ulcer was observed on the anterior wall, and white moss was observed on the uplift. Hyperechoic space was found in the deep submucosal layer of the local stomach, with a cross-section of about 0.1 × 0.1 cm^2^, accompanied by an acoustic shadow in the rear

Computed tomography (CT) was further performed, and the results suggested the gallbladder was slightly larger, the gastric cavity filling was poor, and the gastric antrum was thickened. After careful examination of the film, we found a streaky high-density shadow (approximately 3 cm in length) on the posterior wall of the gastric antrum extending outside the wall, as shown in Fig. 3. The possibility of FBs accompanied by perforation was then considered. Further abdominal X-ray also showed a dense shadow in the duodenal bulb with a length of about 2.7 cm, as shown in Fig. 4. Endoscopic ultrasonography showed that the anterior wall of the duodenal bulb obviously protruded into the cavity. A superficial ulcer was observed on the anterior wall and white moss was observed on the uplift. Hyperechoic space with a cross-section of approximately 0.1 × 0.1 cm was found in the deep submucosal layer of the local stomach, accompanied by an acoustic shadow in the rear as shown in Fig. 5, which led to the consideration of fishbone as the FB. Further questioning of the patient confirmed that she had a history of eating fish soup before she developed abdominal pain.

### Surgical treatment

Abdominal CT examination suggested abscessus and thus, the possibility of perforation was not excluded. It is more difficult to find lesions under laparoscopy and endoscopic treatment is associated with higher risks when perforation is suspected, thus the patient was recommended for exploratory laparotomy. However, the patient and her family refused open surgery and requested endoscopic investigation. However, certain difficulties and complications may arise with endoscopic treatment: 1) the FB stump may not be visible under the endoscope; 2) injury to the adjacent liver and pancreas can occur; 3) after removing the FB, the closed perforation cannot be treated under the endoscope; 4) massive upper gastrointestinal bleeding or perforation can occur during or after surgery. If any of the above conditions occurs, emergency surgery is required. During the operation, we attempted to find the end of the fishbone on the bulges of the ball using FB forceps (MTN-4GF-23, Nanjing minimally invasive), as the tail end could be seen faintly in the abscess. FB forceps were used to clamp the proximal end and a fishbone-like FB with a length of 5.5 cm was pulled out. The ulcer slightly oozed blood and two thrombins were sprayed to stop the bleeding. Fasting as well as acid inhibition and anti-infection medication were prescribed for the patient after surgery.

### Prognosis and follow-up

After treatment, the patient’s abdominal pain disappeared. Endoscopy was performed 1 week later and showed that the ulcer healed well. The patient was discharged and subsequent follow-ups revealed no further abdominal discomfort.

## Discussion and conclusion

Gastrointestinal FBs are defined as any material in the upper gastrointestinal tract that causes symptoms, including impacted food [11]. Currently, there is no unified standard for the definition of chronic upper gastrointestinal FBs. Clinically, the term mostly refers to FBs ingested for various reasons lodged in the digestive tract for more than 1 month, along with mild to moderate discomfort or aggravated discomfort. Due to the lack of obvious symptoms, most patients miss the early treatment window after the ingestion of FBs; therefore, cases of various abscesses resulting from FB perforation are not uncommon. We searched the PubMed database for relevant English articles using the key words “duodenal perforation”, “abscess”, and “foreign body”. A total of 19 articles with the closest match were selected. The FB types, clinical manifestations, diagnostic tools, and treatment strategies for the reported cases were analyzed and are summarized in Table 1.

**Table 1:** Statistics of foreign body types, clinical manifestations, diagnostic tools, and therapeutic strategies in literature review

No.	First author	Foreign body	Clinical symptom	Diagnostic tool	Treatment
1	Jimenez-Fuertes M [12]	fish bone	abdominal pain	ultrasonography, CT	surgery
2	Yao SY [8]	blister pack	backache	CT	laparotomy
3	Glick WA [13]	toothpick	abdominal pain	CT	surgery
4	Lee MK [14]	fish bone	melena	X-ray, endoscopy	endoscopy
5	Jarry J [15]	fish bone	abdominal pain	X-ray, ultrasonography, CT, endoscopy	surgery
6	Chen HK [16]	fish bone	fever, epigastric pain	CT, endoscopy	surgery
7	Jutte E [17]	sewing needle	abdominal pain, nausea	ultrasonography, gastroscopy, CT	laparoscopy
8	Yasuda T [18]	fish bone	abdominal pain	X-ray, CT	laparotomy
9	Su YJ [19]	toothpick	epigastric pain	X-ray, CT	endoscopy, laparotomy
10	Kadowaki Y [20]	fish bone	abdominal pain, fever	ultrasonography, CT	laparotomy
11	Chiang TH [21]	toothpick	abdominal pain, fever	ultrasonography, X-ray	antibiotic
12	Miller G [22]	biliary stent	abdominal pain, nausea, vomiting	CT	laparotomy
13	Goh BK [23]	fish bone	fever	X-ray, CT	laparotomy
14	Newman B [24]	toothpick	abdominal pain, fever	X-ray, ultrasonography, CT	surgery
15	Toyonaga T [25]	needle	diarrhea	X-ray, CT	surgery
16	Perkins M [26]	battery, coin, button, pen nib	fever	X-ray, ultrasonography, CT, endoscopy	endoscopy, antibiotic
17	Drnovsek V [27]	toothpick	abdominal pain	ultrasonography, CT	surgery
18	Archer BD [28]	wooden skewer	Right iliac fossa pain, fever	X-ray	laparotomy, drainage, antibiotic
19	Honaas TO [29]	toothpick	abdominal pain, fever	endoscopy	endoscopy

### FBs

Fish bone [12, 14–16, 18, 20, 23] was the most common FB, followed by toothpicks [13, 19, 21, 24, 27, 29]. Other uncommon types of FB included needles [17, 25], blister packs [8], and wooden skewers [28]. Psychiatric patients ingested a wide variety of FBs (batteries, coins, buttons, pen nibs) [26]. Surprisingly, the FBs also included a bile duct stent [22] implanted in the body to treat disease.

### Clinical manifestations

Among the data we collected, more than half of the patients had abdominal pain [12, 13, 15–22, 24, 27, 29] as the main complaint, followed by fever [16, 20, 21, 23, 24, 26, 29] secondary to abscess. A few patients presented with melena [14], nausea [17, 22], vomiting [22], diarrhea [25], and even non-gastrointestinal symptoms in some cases (backache [8], right iliac fossa pain [28]).

### Diagnostic tools

The diagnostic tools included CT [8, 12, 13, 15–20, 22–27], X-ray [14, 15, 18–21, 23–26, 28], ultrasonography [12, 15, 17, 20, 21, 24, 26, 27], and endoscopy [14–17, 26, 29]. CT was used in almost every case. Endoscopy can be used as both an examination tool and a treatment method. It is worth noting that in some cases, the patient’s results with a standard abdominal X-ray [15, 18, 19] or endoscopic examination were normal, but CT [15–17] examination revealed abnormalities. This is because if the FB is completely surrounded by the mucosa, only gastric mucosa edema and purulent external pressure can be seen under the endoscope. Even with further ultrasound gastroscopy, not all radiopaque FBs produce shadows on ultrasound images [30]. On the other hand, patients often suffer from unspecific symptoms without a clear chief complaint of FB ingestion. All increase the difficulty of endoscopic diagnosis.

As in the present case, though the patient had a long history of unspecific abdominal pain, she was unaware of her history of FB intake at the initial visit as well as the first time the gastroscopy showed bulbous duodenal inflammation with a superficial ulcer. As a result, we did not consider the diagnosis of FB initially and thus the treatment did not result in clinical improvement. However, after careful examination of the abdominal CT image, we suspected that the symptoms might be caused by FBs and further ultrasound gastroscopy confirmed the diagnosis. This served as a reminder for us to make full use of the complementary advantages of different modalities when selecting diagnostic tools.

### Treatment strategies

For both acute and chronic patients, endoscopy is a common treatment choice and is recommended as the first-line treatment. Endoscopy has been reported to achieve more than a 95% success rate in removing FBs [9, 10]. Surgical intervention is primarily indicated in less than 1% of cases [31] and also for patients for whom endoscopy is unsuccessful. Therapeutic strategies in the literature reviewed included endoscopic [14, 19, 26, 29] surgery [8, 12, 13, 15, 17–20, 22–25, 27, 28] and antibiotics [21, 26, 28].

After diagnosis of this patient, we recommended surgery for the patient because the course of the disease was chronic after nearly 3 months. The first gastroscopy failed to show the fish bone and CT revealed perforation of the duodenal bulb and the induction of an abscess. The patient had a good nutritional status, no chronic consumption issues, and no changes in body weight, appetite, feces, or urine. The patient was able to tolerate the trauma and stress of open surgery and general anesthesia. However, the patient and her family strongly requested an attempt at endoscopy. We successfully found the end of the fish bone with endoscopy and pulled out the entire object. No perforation or other complications occurred during follow-up and the patient was determined to be cured and discharged.

Based on our experience with the current case, some suggestions should be taken into consideration. First, the chief complaint, possible contributory factors, dining, and food consumption should be comprehensively investigated. Second, the clinician should be an expert at reading abdominal CT scans and the report of the results issued by auxiliary departments should not be relied upon completely, but only used as a reference. In addition, the role of ultrasound endoscopy in the diagnosis of unclear source lesions in the digestive tract should not be ignored.

Accurate diagnosis is key to successful treatment, for patients suspected of chronic FB ingestion, in addition to careful medical history, careful examination of CT images and further ultrasound endoscopy for gastroscopy evaluation are very helpful. The therapeutic effect is a validation of the correctness of the diagnosis. Endoscopic intervention can be recommended as the first option.

## Data Availability

The datasets used during the current study are available from the corresponding author on reasonable request.

## Abbreviations

FBs: Foreign bodies
CT: Computed tomography
ASGE: American Society for Gastrointestinal Endoscopy
